# P01.52. Comparative study of two bioassays with stressed duckweed and yeast treated with homeopathic preparations

**DOI:** 10.1186/1472-6882-12-S1-P52

**Published:** 2012-06-12

**Authors:** T Jäger, C Scherr, M Simon, P Heusser, S Baumgartner

**Affiliations:** 1 University of Bern, Institute of Complementary Medicine KIKOM, Bern, Switzerland; 2 University of Oldenburg, Oldenburg, Germany; 3 University of Witten-Herdecke, Herdecke, Germany; 4 University of Bern, Bern, Switzerland

## Purpose

In homeopathic basic research the question of the most adequate test systems and apt methodology is still open. We compared two arsenic (As^5+^) stressed bioassays with duckweed (Lemna gibba, a multi-cellular autotrophic organism) and yeast (Saccharomyces cerevisiae, a single-cellular heterotrophic organism) regarding their response towards homeopathic preparations.

## Methods

For duckweed, growth rates of leaf area and leaf number were evaluated. For yeast, growth kinetics were determined by measuring slope, yield, and Et50 (point in time when yield was half maximum) of the sigmoid growth curve. The experiments with duckweed and yeast were performed in parallel (same day, same location and identical homeopathic preparations). After screening 17 substances, three homeopathic preparations (Arsenicum album, nosode, gibberellic acid) were chosen for repeated experimental series. Five independent experiments were conducted for each remedy with both bioassays in parallel. Potency levels used were in the range of 17x–33x for duckweed and 17x–30x for yeast. As controls, unsuccussed and succussed water were used. To examine test system stability, systematic negative control experiments were conducted over the complete experimentation period. All experiments were blinded and randomized.

## Results

The systematic negative control experiments did not yield any significant effects, meaning that the test systems were stable and did not show false positive results. Application of potentized Arsenicum album in the duckweed bioassay yielded growth stimulating effects compared to water controls for the parameters leaf area and leaf number (p<0.001), even using ultramolecular preparations. Potentized nosode preparations also had significant effects on duckweed's leaf area and leaf number (p<0.01). In the yeast system the three homeopathic remedies did not show any significant effects on any growth curve parameter.

## Conclusion

Arsenic stressed duckweed seems to be a promising bioassay for homeopathic basic research. After verifying external reproducibility in independent laboratories, this bioassay might develop into a valuable tool for basic pharmaceutical questions.

